# Medium-Entropy SrV_1/3_Fe_1/3_Mo_1/3_O_3_ with High Conductivity and Strong Stability as SOFCs High-Performance Anode

**DOI:** 10.3390/ma15062298

**Published:** 2022-03-20

**Authors:** Guanjun Ma, Dezhi Chen, Shuaijing Ji, Xinyun Bai, Xinjian Wang, Yu Huan, Dehua Dong, Xun Hu, Tao Wei

**Affiliations:** School of Materials Science and Engineering, University of Jinan, Jinan 250022, China; maguanjun_ujn@163.com (G.M.); 15039772831@163.com (D.C.); jishuaijing_ujn@163.com (S.J.); woderfulgirly2002@163.com (X.B.); wangxinjian_ujn@163.com (X.W.); mse_dongdh@ujn.edu.cn (D.D.); huxun20032004@126.com (X.H.)

**Keywords:** solid oxide fuel cell, medium-entropy SrV_1/3_Fe_1/3_Mo_1/3_O_3_, high conductivity, high stable anode, enhanced fuel catalysis

## Abstract

Perovskite oxides using solid oxide fuel cells (SOFCs) anodes should possess high chemical stability, adequate electronic conductivity and excellent catalytic oxidation for fuel gas. In this work, the medium-entropy SrV_1/3_Fe_1/3_Mo_1/3_O_3_ (SVFMO) with Fe, V and Mo co-existing in the B site of a perovskite structure was fabricated in reducing 5% H_2_/Ar mixed gas: (1) SVFMO demonstrates more stable physicochemical properties when using SOFCs anodes in a reducing environment; (2) the co-existence of Fe, V and Mo in SVFMO forms more small-polaron couples, demonstrating greatly enhanced electronic conductivity. With SVFMO in a porous structure (simulating the porous anode layer), its electronic conductivity can also reach 70 S cm^−1^ when testing at 800 °C in an H_2_ atmosphere; (3) SVFMO with more oxygen vacancies achieves higher catalytic ability for fuel gas, as an SOFCs anode layer demonstrates 720 mW cm^−2^ at 850 °C.

## 1. Introduction

Solid oxide fuel cells (SOFCs) offer great prospects for clean, secure and sustainable energy sources in a variety of applications ranging from small auxiliary power units to large scale power plants [1,2]. The major advantages for SOFCs are the most efficient utilization of various readily available carbon-containing fuels and their superior tolerance to impurities in the fuels [3,4,5]. In the past few decades, perovskite-based and double perovskite oxides have been used as SOFCs anode materials due to their superior fuel flexibility and high electrochemically oxidizing ability [6]. These anode materials with mixed electronic/ionic conducting mechanisms are perceived to hold potential for solving the problems associated with Ni-based anodes, such as anode deactivation by sulfur impurities and anode destruction by re-oxidation or carbon deposition in hydrocarbon fuels [7]. For example, Marina et al. studied the perovskite-structured Sr_1__−__x_La_x_TiO_3_ as potential SOFC anode materials for improved sulfur tolerance [8]. Tao et al. studied that La_1−x_Sr_x_Cr_1−y_Mn_y_O_3_ is active for the electro-oxidation of CH_4_ at high temperatures in the absence of excess steam [9]. Sr_2_MgMoO_6_ as a Ni-free single-phase double perovskite anode demonstrated comparable performance with Ni-based anode in hydrogen, and also revealed low carbon deposition in methane and anti-sulfur poisoning in H_2_/H_2_S [6].

As SOFC anode materials, the general requirements include high electronic conductivity, excellent catalytic activity towards electro-oxidation of fuels, suitable porosity to allow the flow of fuel molecules and reaction products, adequate durability while exposing in fuel gases and low cost [1,2]. According to recently studied perovskite-based oxide anodes, most demonstrated high ionic conductivity, excellent catalytic activity towards hydrocarbon fuels and remarkable re-oxidation tolerance [10]. For perovskite oxides based SOFC anodes, most are fabricated in an air atmosphere. Studies have revealed that some of these perovskite oxide anodes (fabricating in air) being exposed in fuel atmospheres will suffer phase decomposition, forming impurity or durability problems. For example, although double-perovskite Sr_2_CoMoO_6_ as SOFCs anode has revealed high catalytic ability, itself showing apparent phase decomposition under reducing atmospheres such as the creation of secondary phase Sr_2_MoO_4_ [11,12]. La_0.8_Sr_0.2_Cr_0.95_Ru_0.05_O_3_ as SOFCs anode shows apparent phase decomposition in a wet H_2_ atmosphere [13]. SrTiO_3_ doping with Nb exhibits good chemical stability (and high conductivity: 120 S cm^−1^), but shows poor electrocatalytic activity toward H_2_ oxidation [14]. Another critical function for SOFC anode materials is use as current collectors. Herein, the second limitation for most of the perovskite oxides using as SOFC anodes is their low electrical conductivity when compared with Ni-based anodes. For example, at 800 °C and in a reducing anodic atmosphere, the bulk conductivity for dense La_0.75_Sr_0.25_Cr_0.5_Mn_0.5_O_3_ and Sr_2_MgMoO_6_ ceramics are only about 1 S cm^−1^ and 10 S cm^−1^ [6,9,15]. More importantly, when these oxides were fabricated in suitable porosity to allow the flow of fuel molecules and reaction products, a further decrease of conductivity of the porous anodes seemed inevitable. The actual conductivity of the porous perovskite anode materials may be lower by one or two orders of magnitude than that of the porous Ni-yttria stabilized zirconia (YSZ) based anode.

However, a lot of works have proved that the fabrication environment for a small number of perovskite oxides must be in reducing environments. For example, A_2_FeMoO_6_ (A = Ca, Sr and Ba) and Sr_2_VMoO_6_-based perovskite oxides can be fabricated by sintering in reducing atmosphere (5% H_2_/Ar mixture) [16,17]. These perovskite materials fabricating in reducing atmosphere might exhibit higher phase stability when used as SOFCs anodes in fuel gas, which corresponds with higher anode durability (or long-term stability). For anode conductivity, a potential SOFC porous anode material must exhibit an electrical conductivity of ~100 S cm^−1^ or higher at operational temperature [18]. For Ni-based anode materials, its electrical conductivity can reach 2 × 10^5^ S cm^−1^ (pure Ni metal). For porous Ni-YSZ composite anodes, there extrinsic total conductivities are still much higher than 100 S cm^−1^ at 1000 °C [19]. In our previous work, we have proved that Sr_2_FeMoO_6_ (SFMO) can be used as SOFCs anode with high catalytic activity and sufficient ionic conductivity [16]. However, its total bulk conductivity of dense ceramic structure is still lower than SrV_0.5_Mo_0.5_O_3_ (SVMO). It is predictable that the conductivity will further decrease with SFMO anode in suitable porosity. SVMO in reducing atmosphere (such as in H_2_) demonstrated super high electronic conductivity, for example, as high as 2500 S cm^−1^ at 300 °C. However, SVMO as anode material demonstrated moderate catalytic ability for fuel gas due to its low oxide-ion conductivity [17].

It is urgently need that we develop a new anode with high catalytic activity, high conductivity and excellent structural stability when working in a reducing anode environment. Here, in order to make use of the high catalytic ability and high ion conductivity of perovskite-based oxides, the drawbacks such as phase decomposition and low bulk conductivity must be overcome. Based on the higher structural stability of SFMO and SVMO in a reducing atmosphere, we try to further unite the high catalytic ability of SFMO and the high electronic conductivity of SVMO. In this work, single perovskite SrV_1/3_Fe_1/3_Mo_1/3_O_3_ (SVFMO) with V, Fe and Mo as B-site cations was fabricated by reducing 5% H_2_/Ar atmosphere. As SOFCs anode material, SVFMO demonstrated high reduction catalytic ability for fuel gas. In an H_2_ atmosphere, its bulk conductivity can reach as high as 200~500 S cm^−1^ from 30 to 800 °C. With SVFMO in a porous structure (simulating the anode structure), its conductivity can also reach to about 134–70 S cm^−1^ from 30 to 800 °C in H_2_ atmosphere, which is comparable to the conventional Ni-YSZ based anode catalyst. At last, by introducing SVMO and SFMO as contrast materials, SVFMO as SOFCs anode (La_0.9_Sr_0.1_Ga_0.8_Mg_0.2_O_3__−x_ (LSGM) as supporting electrolyte and La_0.8_Sr_0.2_Co_0.8_Fe_0.2_O_3−x_ (LSCF) as cathode) demonstrated the maximum power output, such as 720 mW cm^−2^ at 850 °C, and excellent long-term stability. The superhigh electronic conductivity, excellent catalytic ability and structural stability in reducing environments indicate that SVFMO has great application potential for use as SOFCs anode material.

## 2. Experimental Section

### 2.1. Material Preparations

SVMO and SVFMO powders were synthesized by a conventional solid-state reaction. Stoichiometric ratio of SrCO_3_, V_2_O_5_, Fe_2_O_3_ and MoO_3_ were used as raw materials (Shanghai Macklin Biochemical Co., Shanghai, China), and the final fabricating condition was calcination at 1200 °C in 5% H_2_/Ar for 10 h. SFMO powders were synthesized via sol-gel method with stoichiometric Sr(NO_3_)_2_, Fe(NO_3_)_3_·9H_2_O, (NH_4_)_6_Mo_7_O_24_·4H_2_O and citric acid as starting materials (Sinopharm Chemical Reagent Co., Ltd., Shanghai, China). The final calcination for pure-phase Sr_2_FeMoO_6_ was at 1150 °C in 5% H_2_/Ar for 10 h. LSGM pellets were synthesized by solid-state reaction with La_2_O_3_, SrCO_3_, Ga_2_O_3_, and MgO as starting materials (Shanghai Macklin Biochemical Co., Shanghai, China) and sintered at 1450 °C for 20 h. The LSGM electrolyte synthesized by solid phase method was fixed in a thickness of about 250–300 μm. Ce_0.8_Sm_0.2_O_2_ (SDC) powders were synthesized via sol-gel method with stoichiometric Sm(NO_3_)_3_, Ce(NO_3_)_3_·6H_2_O, and citric acid as starting materials (Sinopharm Chemical Reagent Co., Ltd., Shanghai, China). The final calcination temperature was 800 °C in air for 10 h to obtain the pure phase SDC. La_0.8_Sr_0.2_Co_0.8_Fe_0.2_O_3__−x_ (LSCF) cathode was also synthesized via sol-gel method with stoichiometric La(NO_3_)_3_·6H_2_O, Sr(NO_3_)_2_, Co(NO_3_)_3_·6H_2_O, Fe(NO_3_)_3_·9H_2_O, and citric acid as starting materials (Sinopharm Chemical Reagent Co., Ltd., Shanghai, China). The final calcination temperature was 1050 °C in air for 10 h.

### 2.2. Symmetrical and Single Cells Fabrication

First, the electrode adhesive was prepared by mixing ethyl cellulose and terpinol (Sinopharm Chemical Reagent Co., Ltd., Shanghai, China) at a mass ratio of 1:9. To fabricate a symmetric cell, the slurry prepared by mixing SDC synthesized by sol-gel method and electrode adhesive was deposited onto both sides of the LSGM electrolyte by silk-screen printing and then heated at 1300 °C for 1h to obtain the buffer layer. The anode slurry was deposited onto both sides of the SDC by screen printing and then heated at 950 °C for 5 h in 5% H_2_/Ar 5 h to obtain symmetrical cells. To fabricate single cells, the electrode powders (SVMO, SVFMO, SFMO and LSCF) were grinded with the electrode adhesive to form the electrode slurry. The SDC slurry was deposited onto one side of the LSGM electrolyte by silk-screen printing and then heated at 1300 °C for 1 h to obtain the buffer layer. The cathode slurry (LSCF) was deposited onto another side of the LSGM by silk-screen printing and heated at 1100 °C in air for 5 h. Then, the anode slurry was deposited onto the SDC buffer layer by silk-screen printing and heated to 950 °C in N_2_ for 5 h.

### 2.3. Material Characterizations

The phase purity and crystal structure of the fabricated samples were identified by X-ray diffraction (XRD) using the Philips X’Pert PRO diffractometer (Rigaku, Japan). Temperature-programmed reduction (TPR) measurement was carried out to investigate the reducibility of the samples in a Vodo VDSORB-91x analyzer (QuZhou, China). The heating rate of the sample was 20 °C min^−1^. Thermogravimetric (TG) curves were obtained in a thermal analyzer (STA449F5, Netzsch, Germany) for the thermal analysis. The thermogravimetric sample had a heating rate of 10 °C min^−1^. Thermal expansion coefficient (TEC) was measured with a dilatometer (DIL402 Expedis Classic, Netzsch, Germany) on the rod-shape samples under a flow rate of about 50 mL min^−1^ 5% H_2_/Ar. The elemental composition and valence electron states of the compound were further characterized by X-ray photoelectron spectroscopy (XPS) using the Thermo Fisher X-ray photoelectron spectrometer (Thermo Fisher Scientific, Waltham, MA, USA). The morphology and microstructure of the samples were characterized by scanning electron microscopy (JEOL JSM-6480LV, Tokyo, Japan).

Dense bar (the relative density higher than 90%) and porous bar samples (the relative density about 65%) were prepared to test the conductivities as a function of temperature in H_2_ atmosphere. The relative density of samples was measured via Archimedes drainage method. The as-prepared dense rectangular bars with a dimension of 25.00 × 5.00 × 1.50 mm^3^ were fabricated by uniaxial pressing at 300 MPa and sintering at 1200 °C (SVMO and SVFMO) and 1150 °C (SFMO) for 10 h in 5% H_2_/Ar. The porous bar samples were obtained by compressing the 30% starch + 70% sample (in weight ratio) and sintering at 950 °C for 10 h in 5% H_2_/Ar. The conductivity was subsequently measured using a four-probe method with the count’s measurement multimeter and DC power supply in H_2_ from 30 to 800 °C. Ag wires with a small amount of Ag paste in separate dots were used as current collectors. The area-specific resistance (ASR) of the anodes was measured with a two-electrode symmetrical cell configuration. Electrochemical impedance spectroscopy (EIS) of the cell was measured by an IM6 testing system over the frequency range 0.01–100 kHz. The cell polarization curve was tested using the liner scanning mode of the IM6 testing system at 10 mV s^−1^ from the open circuit voltage to 0 V.

## 3. Results and Discussion

### 3.1. Structure and Thermal Analysis

**Figure 1:**
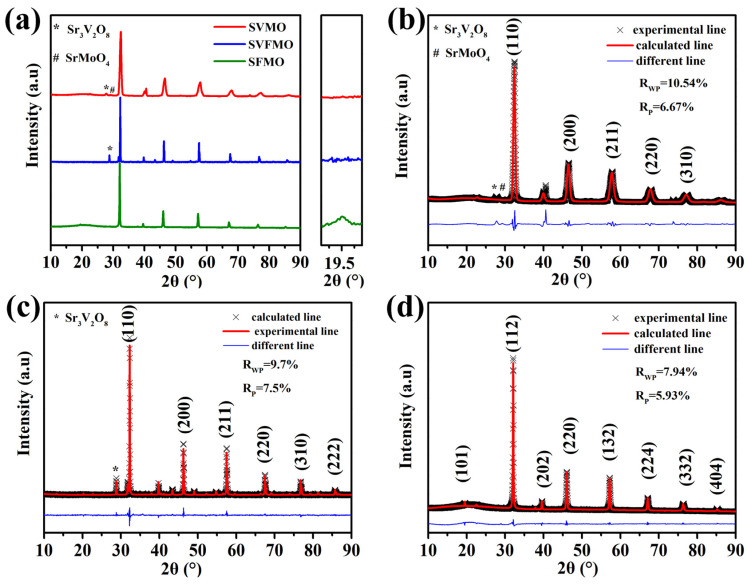
(**a**) XRD patterns of SVMO, SFMO and SVFMO samples tested at room temperature. Rietveld refinement fitting plot for the powder X-ray diffraction pattern of (**b**) SVMO, (**c**) SVFMO and (**d**) SFMO.

Figure 1 shows the room-temperature X-Ray diffraction (XRD) patterns of the as-fabricated materials, which shows that these three samples were successfully synthesized in a 5% H_2_/Ar atmosphere. From the enlarged figure of the XRD diffraction peak, we can see that only SFMO possess an apparent diffraction peak at 19.5°, which is the characteristic peak of double perovskite, meaning that SVMO and SVFMO are in a B-site disordered single perovskite structure [20]. By the structural refinement using Rietveld analysis, the results reveal that a small number of impurities existed in SVMO and SVFMO samples while SFMO was in a pure phase structure. Further, the structure refinement shows that SVMO and SVFMO are in single perovskite structures with B-site cations in a disordered arrangement. SFMO shows the typical double perovskite structure with Fe and Mo elements arranged ordered in the B-site. 

In single perovskite SVFMO, the cations in B positions are disordered. The disordered B-site surely will increase the entropy [21]. To distinguish the entropy system of the as-fabricated materials, the configuration entropy (Sconfig) was calculated using the following equation: $\mathrm{Sconfig}=-R\sum_{i\;=1}^{n}\mathrm{Xi}$ × lnXi (R; the ideal gas constant; n: the number of components in the same position, Xi; the mole fraction of elements in the same position). The calculated Sconfig for SVMO and SFMO are 0.69R, which belong to low entropy. For SVFMO, the higher Sconfig 1.1R corresponds to medium entropy. Here, V, Fe and Mo elements co-doping in the B sites of SVFMO will increase the configuration entropy, which are beneficial to the oxygen ion migration in the lattice [21]. 

**Figure 2:**
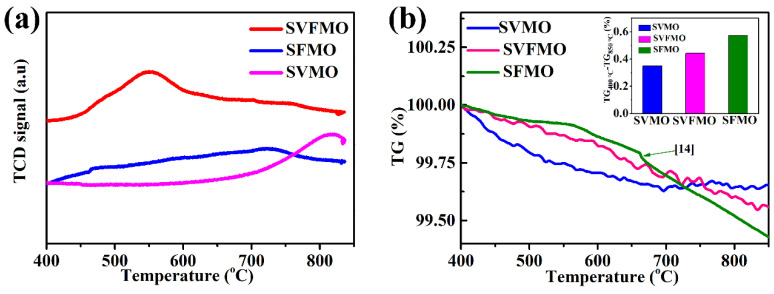
(**a**) H_2_-TPR for the SVMO, SVFMO and SFMO testing from 400 to 850 °C. (**b**) TGA curves of SVMO, SVFMO and SFMO sample testing from 400 to 850 °C in N_2_.

To compare the catalytic ability of the three samples, H_2_-TPR was tested to identify the released oxygen concentration. Figure 2a shows the H_2_-TPR profiles testing between 400 and 850 °C. For SVMO, the apparent reduction peak appears between 750 and 850 °C, which is associated with the change in the oxidation states of V and Mo, such as from V^5+^ to V^4+^, V^3+^, or V^2+^ and Mo^6+^ to Mo^5+^ or Mo^4+^ [17,22]. For SFMO, the replacement of V with Fe demonstrated a decrease of reduction peaks to about 500~600 °C. For SVFMO, the reduction peak shifted to a lower temperature, which means the introduction of Fe reduces the reduction kinetics of V and Mo elements. Herein, the co-existed Fe, V and Mo elements in B sites of SVFMO will accelerate the catalytic ability for an H_2_ reduction. The weight loss of the three samples with increased temperature was tested by thermogravimetry (TG) to study the oxygen loss or the creation of oxygen vacancy. Figure 2b shows the relationship of oxygen loss with the effects of increased temperature. The weight losses of SVMO, SVFMO and SFMO are 0.35%, 0.44% and 0.57% [16] from 400–850 °C (to exclude the effects of moisture), respectively. The weight loss represents an introduction of mobile bulk oxygen vacancies and the reduction of aliovalent cations from high valence (including Mo, V and Fe) to low valence states by a reversible loss of oxygen [16]. Further, as elucidated from the illustration, the weight loss of SVFMO is significantly higher than SVMO in the temperature range. This means more oxygen vacancy was introduced by the loss of oxygen ions from SVFMO, which will facilitate the transport of oxygen ions. 

### 3.2. Electrical Conductivity and XPS Analysis 

**Figure 3:**
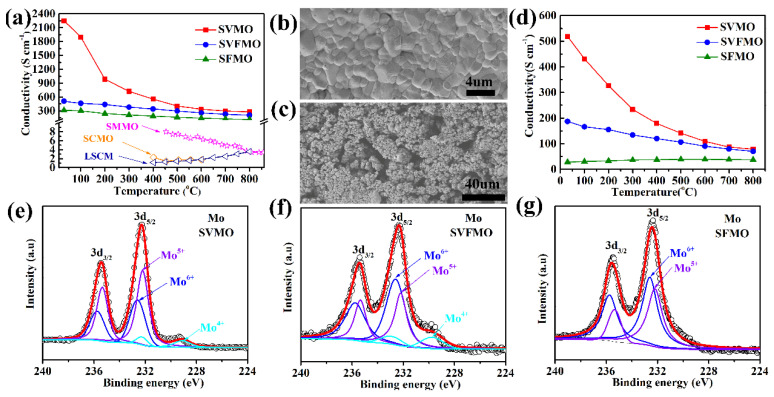
Temperature dependence of electrical conductivity of (**a**) the dense bar and (**d**) the porous bar anode materials. SEM images of SVFMO: (**b**) the dense bar and (**c**) the porous bar. XPS spectra of Mo 3d to evaluate the valence states of Mo elements in (**e**) SVMO, (**f**) SVFMO and (**g**) SFMO.

Conductivity is a critical factor for perovskite oxides using SOFCs electrode materials. The total conductivities of dense SVMO, SVFMO and SFMO bars are tested in the temperature range of 30–800 °C in an H_2_ atmosphere. As shown in Figure 3a, SVMO shows the highest electronic conductivity, such as 2250.9 S cm^−1^ at 30 °C and 279 S cm^−1^ at 800 °C. The conductivity for SVFMO is 508.4–207 S cm^−1^ for testing temperature from 30 to 800 °C. SFMO shows the lowest conductivity, demonstrating 315.3 to 114.2 S cm^−1^ from 30 to 800 °C. For comparison, the total conductivities for other reported typical perovskite anode materials, such as La_0.75_Sr_0.25_Cr_0.5_Mn_0.5_O_3__−__δ_ [23], Sr_2_MgMoO_6_ [6], and Sr_2_CoMoO_6_ [24], are also shown in Figure 3a. We can see that the conductivities of the three reported samples are significantly higher than the compared perovskite anode materials. The morphology of the samples for conductivity testing, with SVFMO as an example, was dense enough as tested by SEM, Figure 3b. However, the SOFCs anodes need to possess a certain degree of porosity to transport fuel gases and exhausts. Moreover, the porous anode materials also need to serve as a current collector. For example, the Ni-YSZ compose anode in porous structure also exhibited excellent conductivity at 800 °C in an H_2_ atmosphere [25]. However, for most of the perovskite anode materials, even in dense structure, their conductivities were one or two orders of magnitude lower than that of the porous Ni-YSZ. Herein, the conductivity of perovskite anodes in porous structure is another critical factor for evaluating their application potential. 70% SVFMO + 30% starch mixture was used to fabricate the porous anode by sintering at 950 °C in 5% H_2_/Ar for 5 h. As shown by the SEM in Figure 3c, the pore structure of the porous SVFMO bar is uniform, very similar to the SOFCs porous anode layer. The conductivities of porous SVMO, SVFMO and SFMO bars were tested as a function of temperature in H_2_. As shown in Figure 3d, the conductivities of porous SVMO, SVFMO and SFMO at 30 °C are 518.5, 186.9 and 28.5 S cm^−^^1^ and at 800 °C are 78.8, 70.1 and 37.9 S cm^−^^1^, respectively. The high conductivities of the porous SVMO and SVFMO can match the demand of an anode layer as current collector. 

The XPS were used to detect the possible valence states of V, Fe and Mo in the three samples. The mixed valence states of the elements were evaluated by fitting the XPS data. First, for the XPS of Fe 2p signals in SVFMO and SFMO (Figure S1), the peaks at the binding energies of 711 and 710.3 eV were assigned to the Fe^3+^ 2p_3/2_ and Fe^2+^ 2p_3/2_ signals, demonstrating the coexistence of Fe^3+^ and Fe^2+^. As shown in Table 1, the proportion of Fe^2+^/Fe^3+^ is 50%: 50% in SVFMO and 43%: 57% in SFMO. As shown in Figure S2 and Table 1, the proportion of high valence-state V^5+^ increased apparently from SVMO (V^5+^ in 39%) to SVFMO (V^5+^ in 47%), which is mainly attributed to the co-doping of low-valence Fe^2+^ and Fe^3+^.

In Figure 3e−g, the peaks at the binding energies of 229.7, 232.2 and 232.6 eV can be assigned to Mo^4+^, Mo^5+^ and Mo^6+^. It is worth noting that Mo^4+^ appears in SVMO and SVFMO, but not in SFMO. In Table 1, there is an apparent increase of Mo^6+^ proportion from SVMO (Mo^4+^ in 6.7%, Mo^5+^ in 58.7%, Mo^6+^ in 34.6%) to SVFMO (Mo^4+^ in 9.4%, Mo^5+^ in 35.5%, Mo^6+^ in 55.1%) and to SFMO (Mo^5+^ in 45.4%, Mo^6+^ in 54.6%). The increased proportion of Mo^6+^ is assigned to the decreased valence states from V^5/4/3+^ to co-existed V/Fe and then to Fe^3/2+^. The V, Fe and Mo with complicated valence states co-existing in the B-site of perovskite structure can form more small-polaron couples as electronic carriers (via the double-exchange mechanism), which is responsible for the high conductivity. For SVMO and SVFMO samples, the superhigh conductivities were attributed to the complicated valence states of V, Fe Mo cations. The increased conductivity was possibly associated with more V, Fe Mo elements in a lower oxidation state to form more small-polaron couples.

### 3.3. EIS Measurements

**Figure 4:**
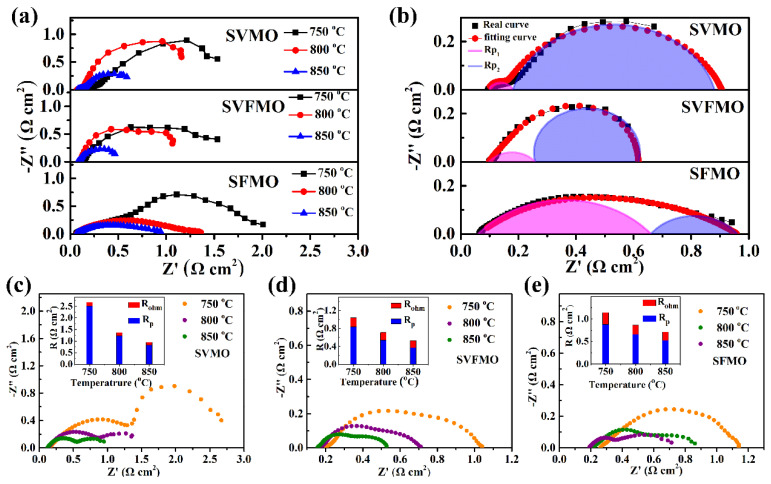
(**a**) EIS of symmetric cells with SVFMO, SVMO and SFMO as working electrodes tested at 750–850 °C in H_2_. (**b**) the fitting EIS (testing at 850 °C) to analyze the contribution of R_P1_ and R_P2_ for symmetric cells (**c**–**e**) the single cells with SVMO, SVFMO and SFMO as anodes and with H_2_ as fuel gas were evaluated by impedance spectra under OCV condition at 750–850 °C (illustrations show the proportions of R_ohm_ and R_P_).

To assess the electrochemical activity of SVMO, SVFMO and SFMO anodes, the symmetrical cells with anode│SDC│LSGM│SDC│anode configurations are used to test the EIS in an H_2_ atmosphere at 750, 800, and 850 °C. First, the three symmetric cells show almost the same ohmic resistance (R_Ohm_). Then, the calculated interfacial polarization resistance (R_P_: the distance between the high and low frequency intercepts) was used to evaluate the catalytic ability of the anode materials. As shown in Figure 4a, the R_P_ decreases gradually with the increase of operating temperature, indicating the thermal activation process. The SVFMO anode shows the lowest R_P_ among the three samples at the same temperature. To further analyze the anode catalytic ability, the impedances tested at 850 °C were fitted as shown in Figure 4b. The equivalent circuit is shown in Figure S3. R_P1_ was contributed to the charge-transfer process at the electrolyte/electrode interface and the electron transfer process accompanying the oxidation of fuel. R_P2_ is associated with the adsorption/diffusion of the fuel gas at the gas/anode interface and the surface diffusion of the intermediate species [26,27]. The fitting data are shown in Table 2. For the SVMO anode, the R_P1_ and R_P2_ were 0.06 and 0.75 Ω cm^2^. This means that the R_P2_ contribution is the main rate-limiting process, which corresponds to the adsorption/diffusion of the fuel gas at the gas/anode interface and the surface diffusion of the intermediate species. However, for the SFMO anode, R_P1_ and R_P2_ were 0.6 and 0.29 Ω cm^2^, suggesting the rate-limiting step was a charge transfer process. For the SVFMO anode, R_P1_ and R_P2_ were 0.16 and 0.36 Ω cm^2^, which means both the enhanced charge-transfer process and adsorption/diffusion for fuel gas. It is noteworthy that the medium-entropy SVFMO with V, Fe and Mo occupying the B-site can help to enhance electrochemical performance. Figure 4c−e shows the typical impedance spectra for single cells measured in open-circuit conditions. With SVMO, SFMO and SVFMO using as SOFCs anodes and testing at 850 °C as examples, the total interfacial polarization resistances (R_P_) are 0.825, 0.522 and 0.371 Ω cm^2^, respectively. The lower R_p_ for SVFMO anode indicates higher H_2_ catalytic ability and faster charge carrier transportation, which was attributed to the forming of more oxygen vacancies and more small-polaron couples. This is also confirmed by TG testing; SVFMO with more weight loss means more oxygen vacancy concentrations were created for hydrogen catalysis.

### 3.4. Single-Cell Performance

**Figure 5:**
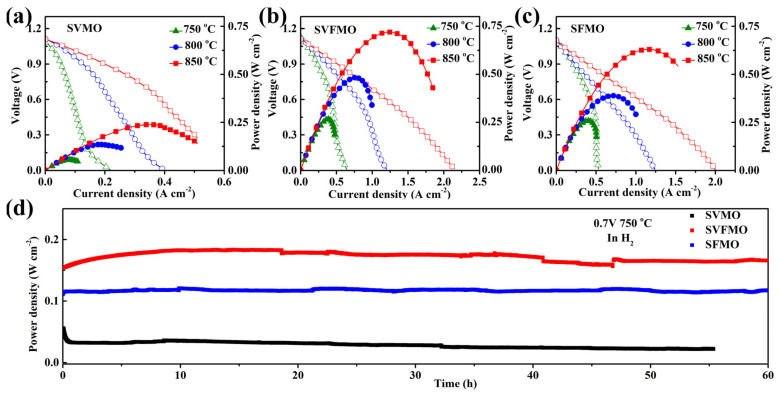
Comparison of the cell voltage and power density testing at 750–850 °C as a function of current densities for single cells with (**a**) SVMO, (**b**) SVFMO and (**c**) SFMO as anodic material. (**d**) Evaluating the long-term stability of the three anode materials testing in 0.7 V at 750 °C.

To evaluate the single-cell performance, LSGM (~250 μm) electrolyte-supported single cells with the configuration of anode|SDC|LSGM|LSCF were fabricated and tested in the temperature range of 750–850 °C. Although the electrolyte-supported single cell hinders us from obtaining the optimum fuel cell performance due to its high ohmic drop, it is useful for us to compare the relative performances of the various electrode materials. The current-voltage (I-V) and current-power (I-P) curves of the single cells are measured and shown in Figure 5. It can be seen from Figure 5a–c that the OCV of the three single cells is in about 1.1V, which is close to the expected values of Nernst potential, demonstrating direct and complete electrochemical oxidation for hydrogen and good sealing with gastight electrolytes. As shown in Figure 5a, SVMO as anode shows the lowest power densities of about 239, 135 and 55 mW cm^−2^ at 850, 800 and 750 °C, respectively. SVFMO as anode exhibits the highest power output of about 720, 484 and 267 mW cm^−2^ at 850, 800 and 750 °C in Figure 5b. SFMO anode exhibits the moderate power output of about 630, 389 and 259 mW cm^−2^ at 850, 800 and 750 °C in Figure 5c. The data are integrated in Table 3. The highest power density for SVFMO anode further demonstrated its improved catalytic activity for fuel gas. The stabilities of the three as-fabricated anodes were evaluated with single cells testing in H_2_ gas at 750 °C for 60 h. As shown in Figure 5d, the three single cells show good stability, which means that the three anode materials fabricated in reducing environment will provide higher tolerance against phase decomposition while working in fuel gas. 

### 3.5. Thermal Expansion and Morphology Analysis

**Figure 6:**
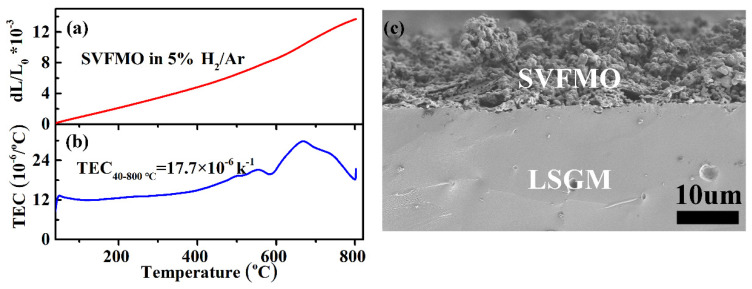
(**a**,**b**)Thermal expansion curves and the calculated TEC for SVFMO dense bar testing between 40–800 °C in 5%H_2_/Ar; (**c**) the local SEM figure showing the good bonding of porous SVFMO electrode layer with dense LSGM electrolyte layer.

For ceramic-based SOFCs, the thermal expansion coefficient of the ceramic components is a critical factor to influence the SOFCs compatibility, thermal stress and even the lifetime [30]. In this work, we have proposed that SVFMO is a potential SOFCs anode material with high conductivity, excellent phase stability and good electrochemical performance. Further, the thermal expansion and TEC of SVFMO were tested from 40 to 800 °C in 5% H_2_/Ar. First, as shown in Figure 6a,b, the expansion curve is almost in a linear relation with increased temperature, which indicated no structural phase transition for SVFMO testing in the proposed temperature range. The increased thermal expansion for SVFMO with increasing temperature is related to the lattice vibrations and the formation of more oxygen vacancies. First, for the lattice vibration, the high-valence states (smaller-ionic size) Mo, Fe and V cations were reduced to lower valence states (larger-ionic size), which cause more pronounced lattice expansion [31]. Second, for oxygen vacancy concentration, the reducing atmosphere and increasing temperature yield more oxygen vacancies, which gives rise to an increase of the repulsive force arising between those mutually exposed cations [32], and resulting in an increase in thermal expansion. The average TEC of SVFMO is 17.7 × 10^−6^ K^−1^, which provided good thermal expansion compatibility with typical SOFC electrolyte materials such as YSZ, LSGM and SDC (TEC: 10.4 × 10^−6^−12.9 × 10^−6^ K^−1^) [17,33,34]. Moreover, the TEC changes appeared between 550–800 °C, agreeing with the oxygen loss calculated from TG and H_2_-TPR results. As shown in Figure 6c, a local SEM picture revealed the good bonding of the porous SVFMO electrode with the dense LSGM electrolyte, no shedding or fault was detected between the electrode/electrolyte interfaces.

## 4. Conclusions

In summary, medium-entropy SVFMO perovskite was fabricated in a reducing environment, demonstrating excellent chemical stability when used in fuel gas as SOFCs anode material. SVFMO with Fe, V and Mo co-existing in a B-site of the perovskite structure increased the configuration entropy and then benefited the oxygen ion migration in the lattice sites, which explained the high catalytic ability for fuel gas. At last, SVFMO with high conductivity, excellent long-term stability and good fuel catalytic activity was demonstrated as a promising SOFCs anode material.

## Figures and Tables

**Table 1:** Comparison of the calculated Mo, V and Fe elements valence ratio in SVMO, SVFMO and SFMO.

Sample	Valence Ratio (%)								
	Mo			V				Fe	
	**Mo^6+^**	**Mo^5+^**	**Mo^4+^**	**V^5 +^**	**V^4+^**	**V^3+^**	**V^2+^**	**Fe^3+^**	**Fe^2+^**
SVMO	34.6	58.7	6.7	39	4.3	44.7	11.9		
SVFMO	55.1	35.5	9.4	47.6	0.9	48.5	3	50	50
SFMO	54.6	45.4						43	57

**Table 2:** The fitting EIS (testing at 850 °C) of R_P1_ and R_P2_ for symmetric cells.

Sample	R_P1_ (Ω cm^2^)	R_P2_ (Ω cm^2^)
SVMO	0.06	0.75
SVFMO	0.16	0.36
SFMO	0.6	0.29

**Table 3:** Power density (mW cm^−2^) for single cells with SVMO, SVFMO and SFMO as anodic material at different temperatures.

Sample	750 °C	800 °C	850 °C
SrV_0.5_Mo_0.5_O_3_	55	135	239
SrV_1/3_Fe_1/3_Mo_1−3_O_3_	267	484	720
Sr_2_FeMoO_6_	259	389	630
Ba_2_FeMoO_6_ [16]	397	521	605
Sr_2_TiMoO_6_ [28]	141	317	505
La_0.4_Sr_0.4_TiO_3_-Ce_0.9_Gd_0.1_O_1.95_ [29]	100	170	250

## Data Availability

The data that support the findings of this study are available from the corresponding author upon reasonable request.

## Supplementary Materials

The following supporting information can be downloaded at: https://www.mdpi.com/article/10.3390/ma15062298/s1, Figure S1: XPS of Fe 2p for SVFMO and SFMO anode; Figure S2: XPS of V 2p for SVMO and SVFMO anode; Figure S3: The equivalent circuit for the fitting EIS of symmetric cells (testing at 850 °C).
