# Supplementary material for: Medium-Entropy SrV1/3Fe1/3Mo1/3O3 with High Conductivity and Strong Stability as SOFCs High-Performance Anode
Source: Materials (Basel). 2022 Mar 20;15(6):2298. doi: 10.3390/ma15062298 (PMC8953221; doi:10.3390/ma15062298)
Supplement: Supplementary file 1 [file materials-15-02298-s001.zip › materials-1614421-supplementary.pdf]

*Supplementary Materials*

# Medium-Entropy $SrV_{1/3}Fe_{1/3}Mo_{1/3}O_3$ with High Conductivity and Strong Stability as SOFCs High-Performance Anode

**Guanjun Ma, Dezhi Chen, Shuaijing Ji, Xinyun Bai, Xinjian Wang, Yu Huan,* Dehua Dong, Xun Hu and Tao Wei***

School of Materials Science and Engineering, University of Jinan, Jinan 250022, China; maguanjun_ujn@163.com (G.M.); 15039772831@163.com (D.C.); jishuaijing_ujn@163.com (S.J.); woderfulgirly2002@163.com (X.B.); wangxinjian_ujn@163.com (X.W.); mse_dongdh@ujn.edu.cn (D.D.); huxun20032004@126.com (X.H.)

* Correspondence: mse_huany@ujn.edu.cn (Y.H.); mse_weit@ujn.edu.cn (T.W.)

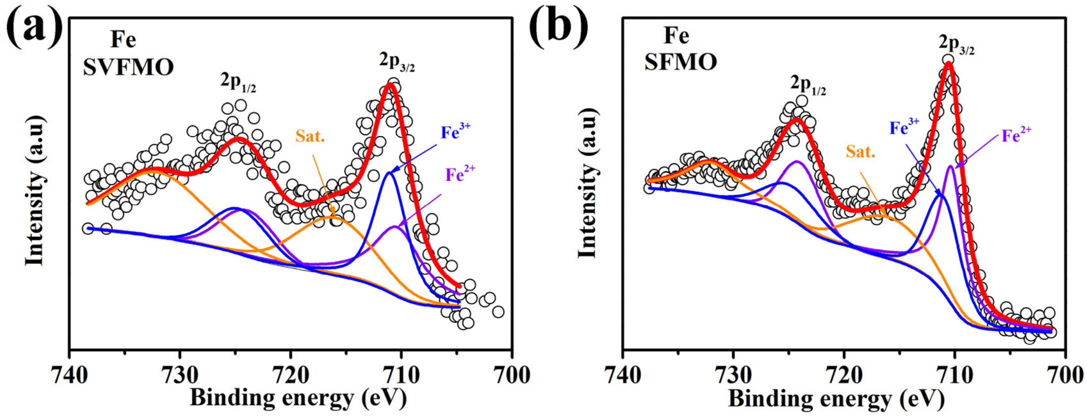


**Figure S1.** XPS of Fe 2p for SVFMO and SFMO anode.

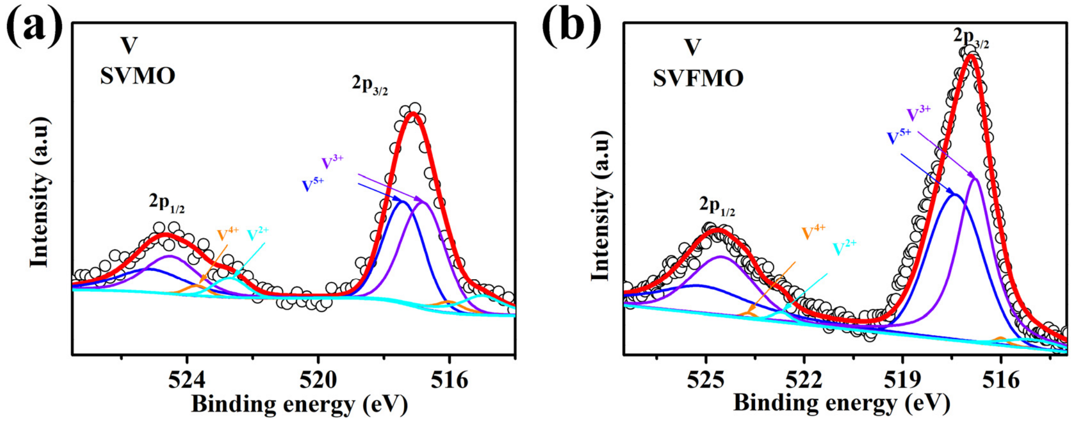


**Figure S2.** XPS of V 2p for SVMO and SVFMO anode.

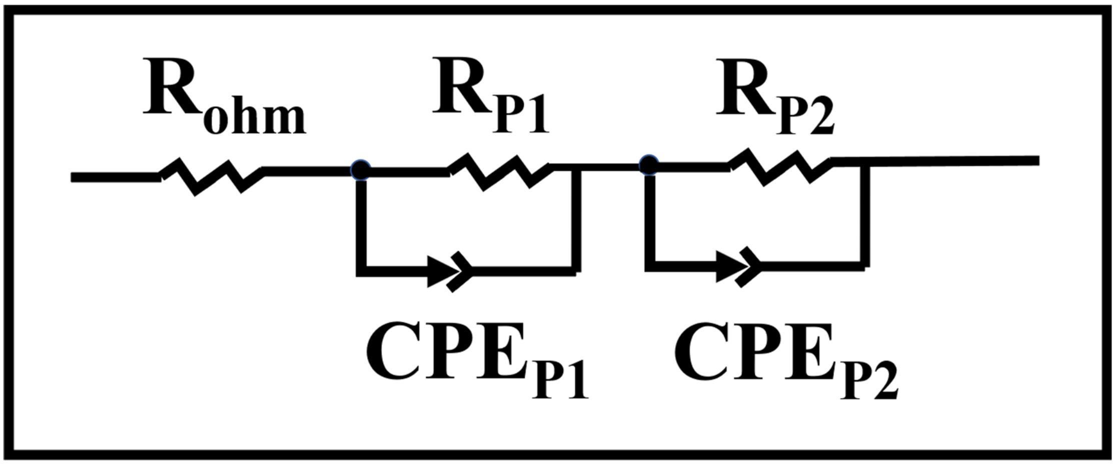

**Figure S3.** The equivalent circuit for the fitting EIS of symmetric cells (testing at 850).
